# Prospective, randomized comparison of gadopentetate and gadobutrol to assess chronic myocardial infarction applying cardiovascular magnetic resonance

**DOI:** 10.1186/s12880-015-0099-3

**Published:** 2015-11-17

**Authors:** Andre Rudolph, Daniel Messroghli, Florian von Knobelsdorff-Brenkenhoff, Julius Traber, Johannes Schüler, Ralf Wassmuth, Jeanette Schulz-Menger

**Affiliations:** Working Group CMR, Experimental and Clinical Research Center, a joint cooperation between the Charité Medical Faculty and the Max-Delbrück Center for Molecular Medicine, Lindenberger Weg 80, 13125 Berlin, Germany; Dept. of Cardiology and Nephrology, HELIOS-Kliniken Berlin Buch, Schwanebecker Chaussee 50, 13125 Berlin, Germany; Department of Congenital Heart Disease and Pediatric Cardiology, Deutsches Herzzentrum Berlin, Berlin, Germany

**Keywords:** Cardiovascular Magnetic Resonance, Contrast media, Gadobutrol, Gd-DTPA, Chronic myocardial infarction, Late gadolinium enhancement

## Abstract

**Background:**

We hypothesized that the contrast medium gadobutrol is not inferior compared to Gd-DTPA in identifying and quantifying ischemic late gadolinium enhancement (LGE), even by using a lower dose.

**Methods:**

We prospectively enrolled 30 patients with chronic myocardial infarction as visualized by LGE during clinical routine scan at 1.5 T with 0.20 mmol/kg Gd-DTPA. Participants were randomized to either 0.15 mmol/kg gadobutrol (group A) or 0.10 mmol/kg gadobutrol (group B). CMR protocol was identical in both exams.

LGE was quantified using a semiautomatic approach. Signal intensities of scar, remote myocardium, blood and air were measured. Signal to noise (SNR) and contrast to noise ratios (CNR) were calculated.

**Results:**

Signal intensities were not different between Gd-DTPA and gadobutrol in group A, whereas significant differences were detected in group B. SNR of injured myocardium (53.5+/−21.4 vs. 30.1+/−10.4, *p* = 0.0001) and CNR between injured and remote myocardium (50.3+/−20.3 vs. 27.3+/−9.3, *p* < 0.0001) were lower in gadobutrol. Infarct size was lower in both gadobutrol groups compared to Gd-DTPA (group A: 16.8+/−10.2 g vs. 12.8+/−6.8 g, *p* = 0.03; group B: 18.6+/−12.0 g vs. 14.0+/−9.9 g, *p* = 0.0016).

**Conclusions:**

Taking application of 0.2 mmol/kg Gd-DTPA as the reference, the delineation of infarct scar was similar with 0.15 mmol/kg gadobutrol, whereas the use 0.10 mmol/kg gadobutrol led to reduced tissue contrast.

**Trial registration:**

The study had been registered under EudraCT Number: 2010-020775-22. Registration date: 2010.08.10

## Background

Myocardial infarction is a leading cause of mortality worldwide. Accurate assessment of infarct size and morphology is important for clinical decision making in a lot of clinical settings [1, 2]. Currently, late gadolinium enhancement cardiac magnetic resonance (LGE-CMR) is a well-established and accurate method to assess scar morphology [3] and is widely used in clinical CMR [4, 5]. Until now, Gd-DTPA, a linear ionic gadolinium chelate, is the extracellular contrast agent that is mostly used for detection of myocardial LGE and is commonly administered as double dose (0.2 mmol/kg). After the administration of Gd-DTPA, cases of nephrogenic systemic fibrosis (NSF) in patients with renal failure have been reported, leading to a new risk classification of contrast media [6]. Whereas the detailed mechanism of NSF is not known, it could be shown in animal experiments that the tissue gadolinium distribution is altered in case of renal impairment [7].

Gadobutrol is a macrocyclic nonionic gadolinium chelate with a higher T_1_ relaxivity compared to Gd-DTPA (gadobutrol: r1 = 4.7 mmol^−1^s^−1^; Gd-DTPA: r1 = 3.9 mmol^−1^s^−1^, at 1.5 Tesla, in human blood plasma, at 37 °C) [8]. The binding profile is known to be more stable, thus through to have a lower risk of NSF. Only three controversially discussed cases of NSF were reported after the use of gadobutrol worldwide until now [9, 10]. Therefore gadobutrol is classified as a low risk contrast-medium. This and the higher relaxivity are driving forces for further evaluations in cardiovascular settings.

Today, there are only limited data available analyzing the influence of contrast agents with different relaxivities on LGE quantification. In this study, we hypothesized that gadobutrol, which is characterized by a higher relaxivity compared to Gd-DTPA, is not inferior to Gd-DTPA in identifying and quantifying ischemic LGE, even when using a lower dose.

## Methods

### Patients’ enrollment

Between September 2010 and December 2011, patients with a history of a chronic myocardial infarction and LGE as assessed during a clinical CMR scan using 0.20 mmol/kg Gd-DTPA were screened and asked for participation in a second study-related CMR scan. All included patients gave written informed consent and were scheduled for a second CMR examination afterwards. The study had been registered under EudraCT number: 2010-020775-22 and was accordingly approved by the ethical board of the city of Berlin (LAGESO).

The sample size of this randomized prospective trial was calculated based on a power calculation. The assumption was, that for a two-sided alpha of 5 % and a power of 80 % based on a delta of 0.15, 15 patients per dose gadobutrol have to be included.

Patients were randomized for either 0.15 mmol/kg (group A) or 0.10 mmol/kg (group B) gadobutrol. All patients were in normofrequent sinus rhythm.

#### Inclusion criteria

- between 18 and 80 years of age
- chronic myocardial infarction (>3 months old) based on coronary artery disease (CAD), detected by invasive angiography
- successfully performed clinical LGE CMR study within the last 4 weeks
- evidence of single myocardial infarction on LGE CMR with “hyper-enhancement” involving at least two contiguous short-axis slices
- written informed consent

#### Exclusion criteria

- severe arrhythmia, atrial fibrillation
- history of moderate or severe impairment of renal function (GFR < 60 ml/min)
- additional myocardial infarction or acute coronary syndrome during the last 4 weeks
- general contraindications for CMR

#### Image acquisition

CMR was performed on a 1.5Tesla cardiac-dedicated clinical MR system (Avanto, Siemens Healthcare, Erlangen, Germany) using a 12-channel coil. Identical CMR protocols were applied for both examinations.

##### LGE

LGE was assessed in all patients starting 13 min after contrast administration in a short axis stack by using a 2D inversion recovery gradient echo sequence (slice thickness 8 mm, no gap, TE 5.0 ms, FA 30°, matrix 256×192, field of view 350×262mm) with an inversion time (TI) adjusted to null signal from normal myocardium. In case of questionable bright signal in the myocardium during the scan, we changed the read out or acquired a perpendicular slice to exclude artefacts. Slice position was identical as for cine imaging using a single slice single breathold approach.

##### T_1_ quantification

T_1_ quantification was performed with a Modified Look-Locker Inversion-recovery (MOLLI) sequence [11], acquired in a mid-ventricular short-axis slice before and 1, 3, 5, 9, 20 min after contrast administration. Multiple post-contrast measurements were performed to show the optimal time point of image acquisition after contrast administration. Imaging parameters were: non-selective inversion pulse, steady-state free precession single-shot read out in mid-diastole, field of view 223 × 320 mm, matrix 174 × 192, slice thickness 8 mm, TE 1.08 ms, FA 35°, bandwidth 1002Hz/Px, minimum inversion time of 100 ms, maximum inversion time of 3600 ms.

#### Image analysis

##### Function

For analyzing LV function and volumes, the endocardial and epicardial contours were manually drawn in systole and diastole using dedicated software (CMR^42^, circle, Calgary, Canada). LV mass was calculated as total myocardial volume multiplied by the specific gravity of the myocardium (1.05 g/ml). LV mass and LV end diastolic volume were indexed to height.

##### LGE

The readers were blinded to the group assignment. For quantification of LGE a semiautomatic gray-scale threshold technique was performed as published previously [12]. Areas of LGE were defined as a signal intensity of more than 6 standard deviations (SD) above the mean of remote myocardium.

Signal intensities of scar tissue, remote myocardium, blood and air were measured by drawing free-hand ROIs of approximately 10–20 pixels in each LGE short axis stack. Reproducible locations were achieved by using anatomical landmarks in dedicated software (CMR^42^, circle international, Calgary Canada). For signal-to-noise ratio (SNR) and contrast-to-noise ratio (CNR) the following sequations were used [13]:

$$ \mathrm{S}\mathrm{N}\mathrm{R} = \mathrm{mean}\ \mathrm{signal}\ \mathrm{of}\ \mathrm{R}\mathrm{O}\mathrm{I}/\mathrm{standard}\ \mathrm{deviation}\ \mathrm{of}\ \mathrm{background}\ \mathrm{noise}.\ \mathrm{C}\mathrm{N}\mathrm{R}\ \left(\mathrm{between}\ \mathrm{tissue}\ \mathrm{A}\ \mathrm{and}\ \mathrm{tissue}\ \mathrm{B}\right) = \mathrm{S}\mathrm{N}\mathrm{R}\ \left(\mathrm{A}\right)\hbox{--} \mathrm{S}\mathrm{N}\mathrm{R}\ \left(\mathrm{B}\right). $$

##### T_1_ quantification

**Fig. 1:**
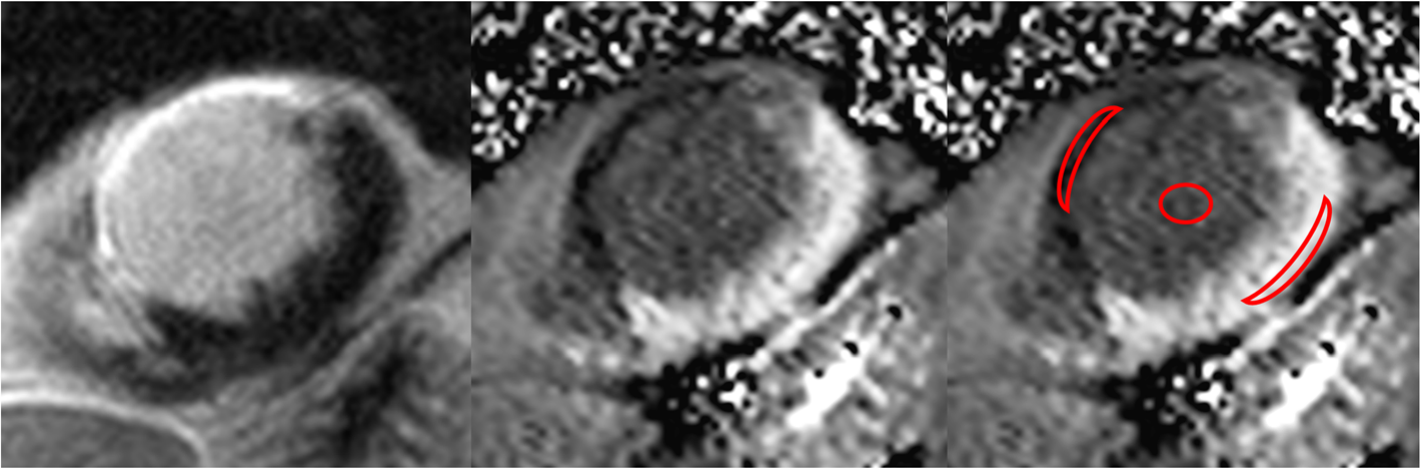
T_1_-Mapping. LGE-image in short axis view (left) with corresponding T_1_-map (center and right) show a transmural anteroseptal myocardial infarction. ROIs (infarct region, remote myocardium and blood pool) were drawn on the map (right)

T_1_ maps were constructed offline using MRmap [14, 15] a customized software program written in Interactive Data Language (IDL; RSI International, Boulder, CO, USA). The position of the source images was initially manually adjusted to correct for potential misregistration. A curve fit of the MOLLI source images was then performed, with automatic calculation of T_1_ values for each pixel. A T_1_ parametric map was subsequently generated and used for further analysis. The parametric maps were evaluated in CMR^42^. Freehand ROI’s of approximately 10–20 pixels were placed in scar tissue, remote myocardium and blood for measuring the averaged T_1_ time in this tissue. Reference for scar delineation was the LGE image in the same plane (Fig. 1).

#### Interobserver variability

To test inter-observer variability in LGE quantification, two readers who were blinded to each other’s results analyzed all examinations.

#### Statistical analysis

Statistical analyses were performed using SAS 9.2 (SAS Institute Inc., Cary, NC, USA) and SPSS 21.0 for windows (Chicago, Il, USA). Data are presented as mean ± standard deviation. Statistical tests were performed using non-parametric methods due to the low sample size within each group. Continuous data were compared using the Wilcoxon rank sum tests. Paired data were compared using Wilcoxon signed rank tests. Categorical data were compared using Fisher's exact test. Statistical tests were considered significant with the two-sided p < 0.05. Spearman correlation coefficient’s were used to determine observer-related variability. Boxplots were generated with SPSS and show median (line in the middle), 1st quartile (bottom of box), 3rd quartile (top of box), lowest case within 1.5 times IQR (bottom whisker), highest case within 1.5 times IQR (top whisker) and outliers.

## Results

We screened 252 patients and identified 51 who met our criteria. 30 patients agreed to participate in the trial and were prospectively enrolled in the study and randomized to either 0.15 mmol/kg gadobutrol (group A) or 0.10 mmol/kg gadobutrol (group B). 21 patients refused participation due to a lack of motivation or fear of repeated contrast administration. There were no significant differences between groups relating age, scar amount and signal intensities for control agent Gd-DTPA (Table 1). The time duration between both scans was at maximum 30 days. We took the history again to check for clinical events. In none of the patients a clinical event was existing.

**Table 1:** Patient’s characteristics

	Group A	Group B
Male/female	15/0	15/0
Age (years)	70.20 ± 4.70	63.50 ± 10.70
Scar amount (g)	16.77 ± 10.18	18.63 ± 11.98
SNR scar	45.51 ± 22.56	53.45 ± 21.40
CNR between scar tissue and remote myocardium	41.76 ± 21.24	50.27 ± 20.30

No complications related to contrast administration were observed. Infarcts were detectable in all patients.

### Comparison of signal intensities and LGE amount

**Fig. 2:**
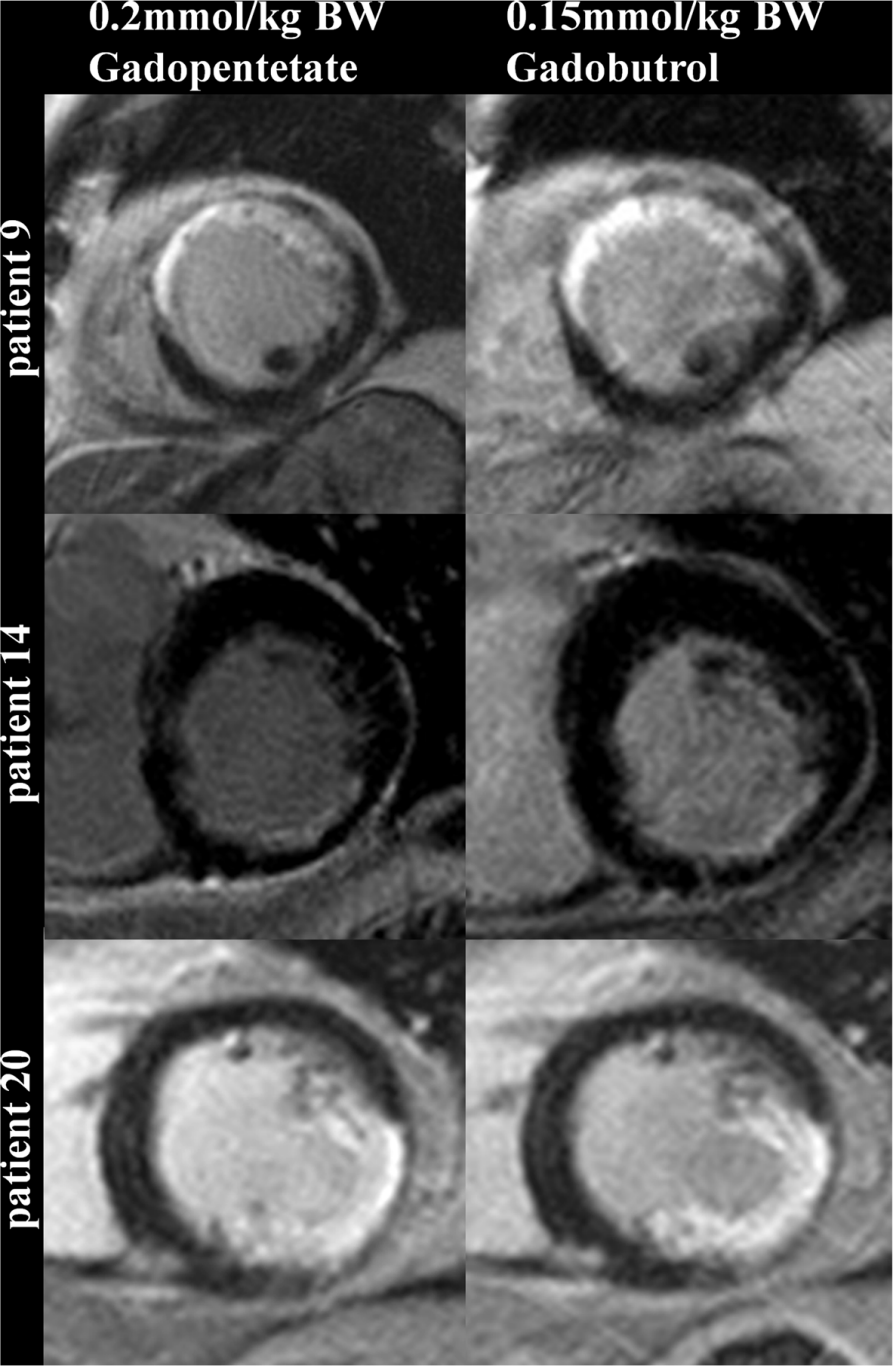
LGE (short axis view) example images from group A. Left: after 0.2 mmol/kg BW Gd-DTPA. Right: after 0.15 mmol/kg BW gadobutrol

**Fig. 3:**
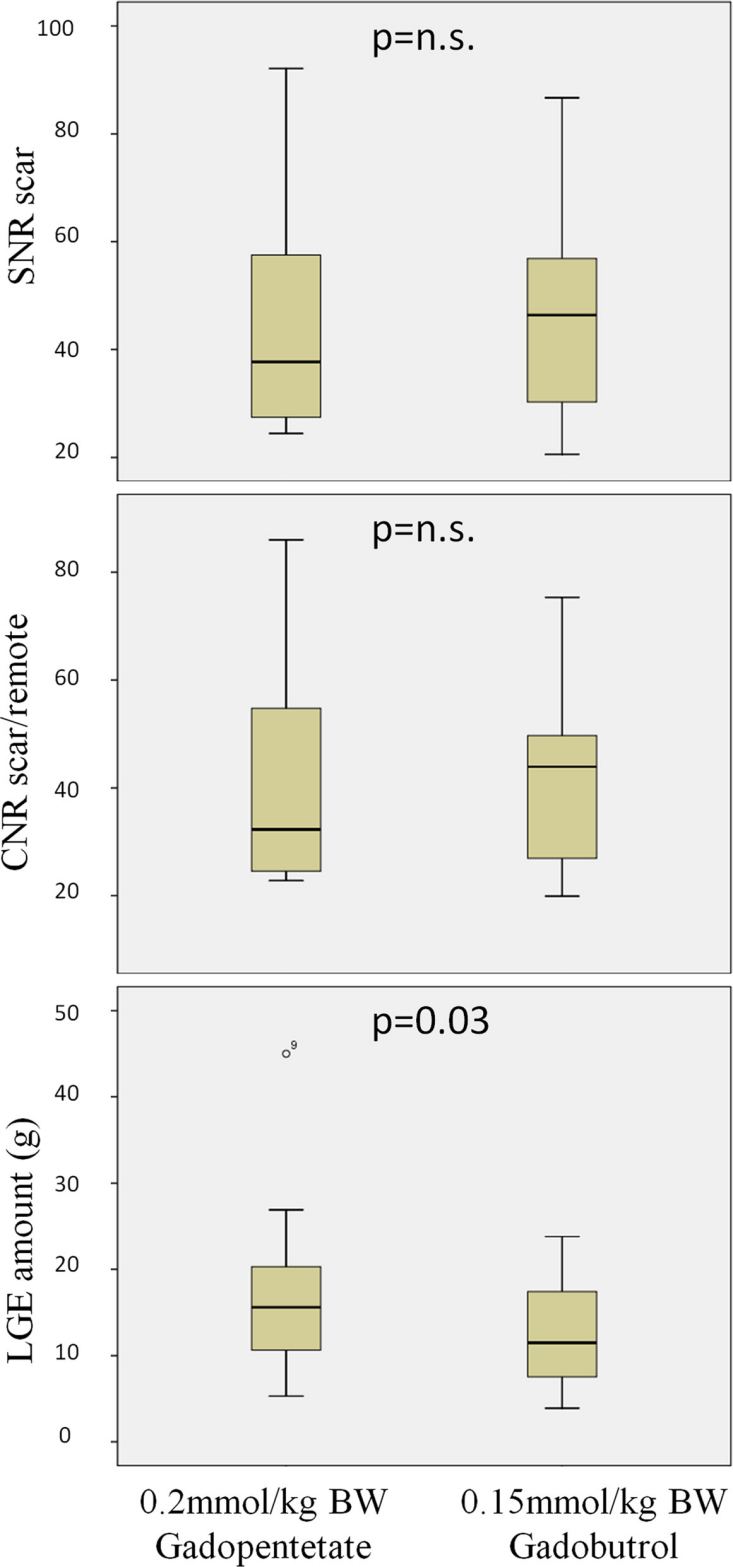
Boxplots showing SNR-, CNR and LGE amount in group A. Left: after 0.2 mmol/kg BW Gd-DTPA. Right: after 0.15 mmol/kg BW gadobutrol

In group A signal intensities showed no differences between Gd-DTPA and gadobutrol (Table 2, Figs. 2 and 3). Amount of LGE was lower in gadobutrol, the absolute difference was small, but reached statistical significance (*p* = 0.03).

**Table 2:** Signal intensities and LGE quantification in group A

	Gd-DTPA (0.2 mmol/kg)	Gadobutrol (0.15 mmol/kg)	p-value
SNR of scar tissue	45.51 ± 22.56	47.04 ± 19.78	>0.9999
CNR between scar tissue and remote myocardium	41.76 ± 21.24	42.83 ± 17.31	>0.9999
SNR of blood	43.97 ± 16.32	44.37 ± 16.20	0.6698
CNR between scar tissue and blood	1.53 ± 17.18	2.65 ± 14.02	0.5153
Amount of LGE (g)	16.77 ± 10.18	12.84 ± 6.79	0.0300

**Fig. 4:**
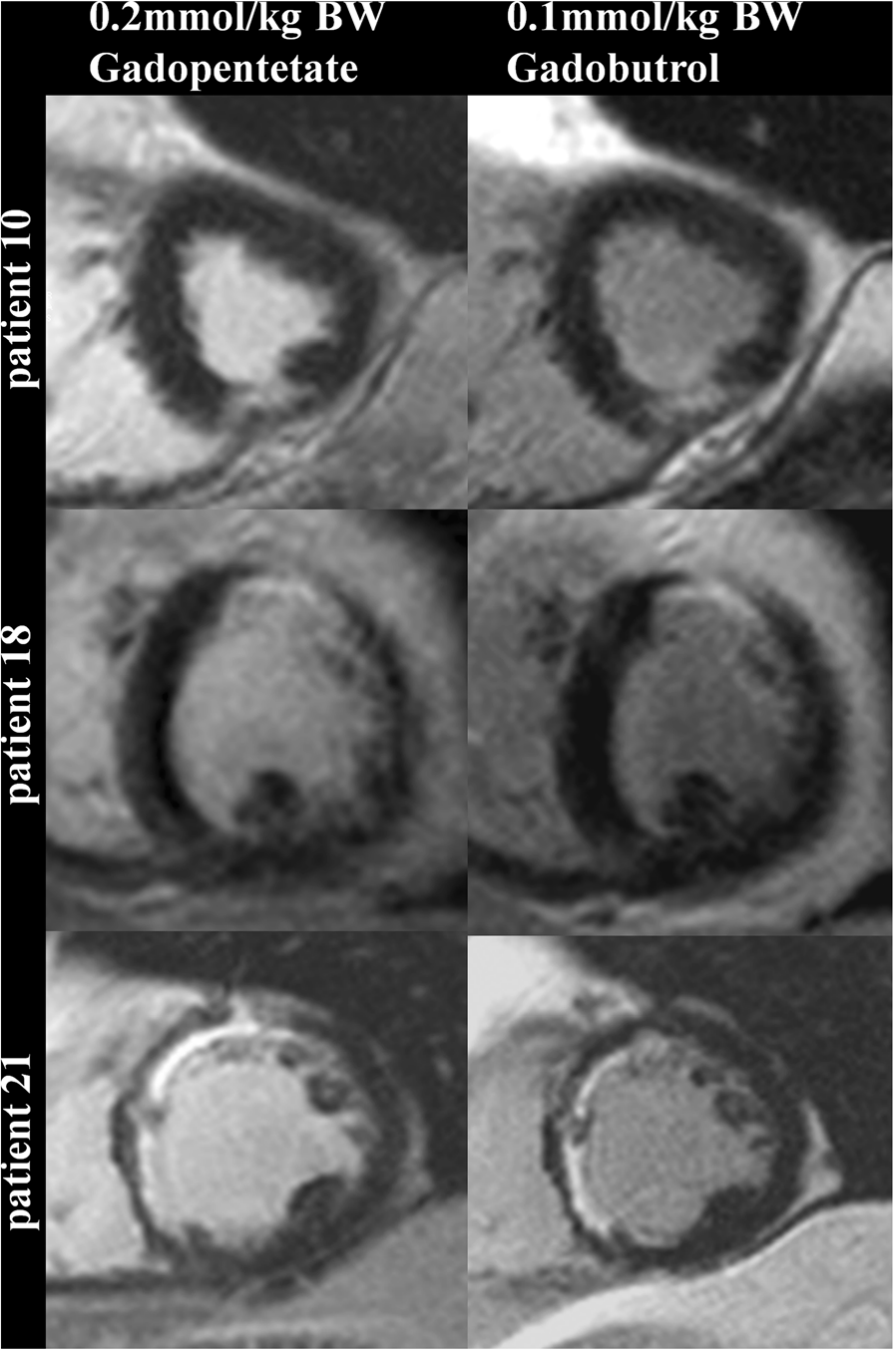
LGE (short axis view) example images from group B. Left: after 0.2 mmol/kg BW Gd-DTPA. Right: after 0.1 mmol/kg BW gadobutrol

**Fig. 5:**
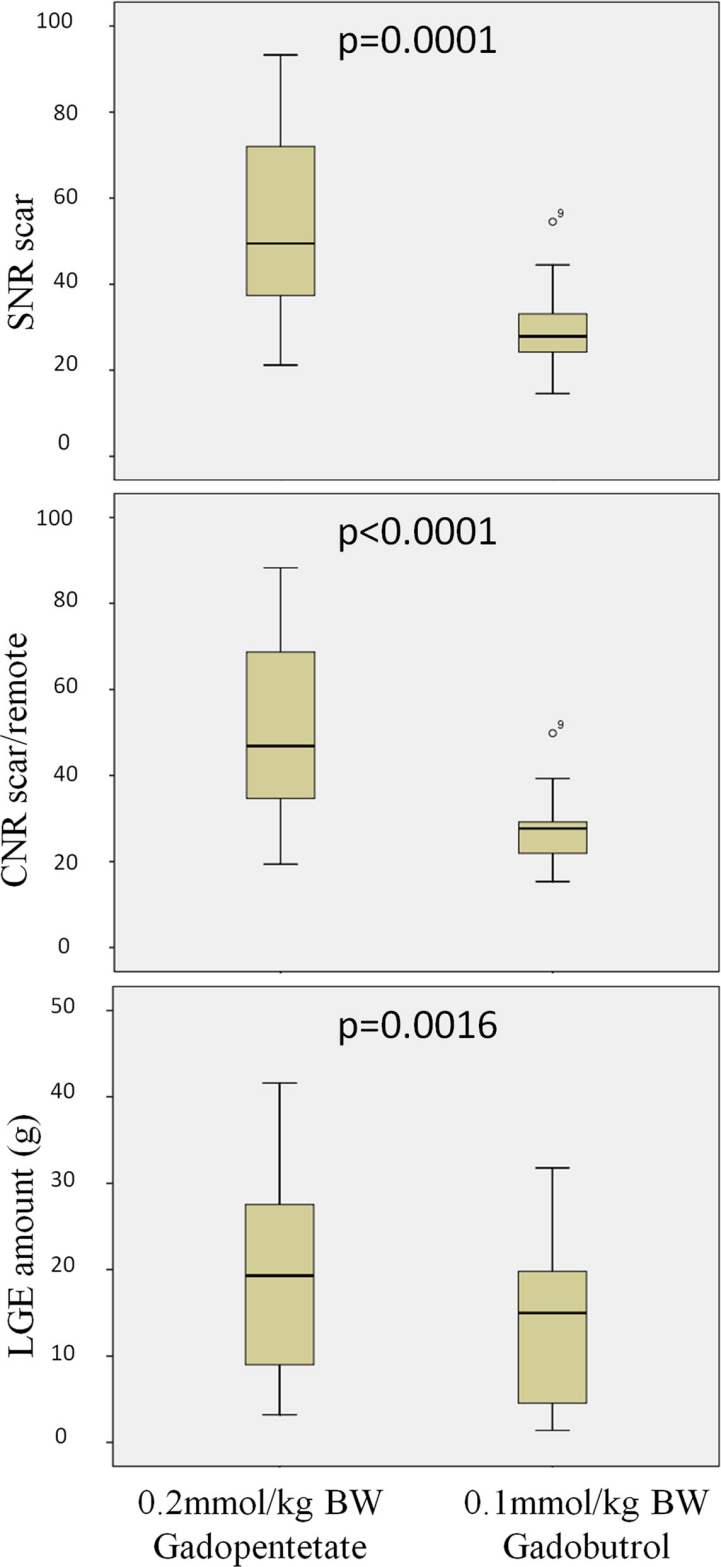
Boxplots showing SNR-, CNR and LGE amount in group B. Left: after 0.2 mmol/kg BW Gd-DTPA. Right: after 0.1 mmol/kg BW gadobutrol

In group B signal intensities showed significant differences between Gd-DTPA and gadobutrol. In particular SNR of injured myocardium and CNR between injured and remote myocardium were significantly lower in gadobutrol resulting in a significant smaller scar size as defined by the amount of LGE (Table 3, Figs. 4 and 5).

**Table 3:** Signal intensities and LGE quantification in group B

	Gd-DTPA (0.2 mmol/kg)	Gadobutrol (0.1 mmol/kg)	p-value
SNR of scar tissue	53.45 ± 21.40	30.11 ± 10.36	0.0001
CNR between scar tissue and remote myocardium	50.27 ± 20.30	27.32 ± 9.28	<0.0001
SNR of blood	48.57 ± 17.69	19.84 ± 6.33	<0.0001
CNR between scar tissue and blood	4.89 ± 12.96	10.29 ± 9.26	0.0554
Amount of LGE (g)	18.63 ± 11.98	14.03 ± 9.92	0.0016

### Interobserver Variability of LGE quantification

Interobserver variability was low and without differences between the contrast agents in both groups (Table 4).

**Table 4:** Spearman’s correlation coefficients between the two readers

	Gd-DTPA	Gadobutrol
Group A	0.85	0.93
Group B	0.91	0.94

**Table 5:** T_1_ values (ms) in group A (*n* = 6)

Time after contrast administration	Gd-DTPA (0.2 mmol/kgKG)	Gadobutrol (0.15 mmol/kgKG)	p-value
Native			
myocardium	909 ± 199	990 ± 117	0.17
scar	1033 ± 226	978 ± 98	0.92
blood	1407 ± 42	1402 ± 62	0.53
1 min			
myocardium	248 ± 38	250 ± 36	0.92
scar	207 ± 28	210 ± 36	0.75
blood	134 ± 18	144 ± 15	0.35
3 min			
myocardium	305 ± 50	311 ± 23	0.75
scar	227 ± 18	241 ± 36	0.35
blood	181 ± 23	200 ± 17	0.25
5 min			
myocardium	361 ± 57	360 ± 18	0.92
scar	275 ± 31	285 ± 48	0.46
blood	215 ± 21	248 ± 52	0.08
9 min			
myocardium	416 ± 61	405 ± 22	0.6
scar	300 ± 50	308 ± 47	0.35
blood	260 ± 28	265 ± 21	0.6
20 min			
myocardium	472 ± 63	468 ± 35	0.89
scar	351 ± 67	361 ± 64	0.5
blood	330 ± 42	337 ± 26	0.5

### T_1_ mapping

For technical reasons we were able to perform a pairwise analysis in only six patients of group A. Interestingly, in this subgroup we found no significant differences between both contrast media regarding T_1_ times of the different tissues at different time- points after contrast administration (Table 5).

The drop-out rate was high due to two main-aspects: I) The applied sequence was a first generation one as described in the method section. II) The most frequent limitation was the registration failure of the raw images especially in regions of wall thinning respectively the infarct area.

## Discussion

In the present study we compared the depiction of chronic myocardial infarction using two different contrast media and different doses in a prospective randomized setting. The null hypothesis of our study was, that the infarct volume with gadobutrol is not equivalent to the infarct volume with the control agent. This was confirmed by our results. The main results are the following:

I) Scar was detectable with each contrast media and dose. II) The use of 0.15 mmol/kg BW (body weight) gadubutrol led to similar results compared to 0.2 mmol/kg BW Gd-DTPA regarding signal intensity and contrast whereas the application of 0.1 mmol/kg gadobutrol led to a significant poorer delineation of scar tissue. III) Both doses gadobutrol led to a smaller infarct size taking 0.2 mmol/kg BW Gd-DTPA as the reference. IV) Observer variability of LGE quantification was independent from type and dose of contrast agent.

Although our sample size was small, but defined in a random setting, we could confirm in part the data by Durmus et al., who compared 0.15 mmol/kg BW gadobutrol with 0.2 mmol/kg BW Gd-DTPA in 20 patients with myocardial infarction [16]. They showed that gadobutrol led to similar infarct size and CNR between scar and remote myocardium compared to Gd-DTPA. In contrast to our results, CNR between scar and blood even increased with gadobutrol in their study, whereas we observed no significant difference in group A between gadubutrol and Gd-DTPA regarding SNR of blood as well as CNR between scar and blood. Our finding are supported by the quantitative parametric T1-mapping results as presented in a subgroup.

DeCobelli et al. compared gadobutrol with Gd-DTPA in patients with positive LGE regardless of its etiology [17]. They showed that 0.1 mmol/kg BW gadobutrol is as effective as 0.2 mmol/kg BW Gd-DTPA regarding signal intensities and quantification of injured tissue. Our data (group B) are different to these results. In our study 0.1 mmol/kg BW gadobutrol led to poorer delineation of infarct scar and reduced amount of LGE as found by both readers. A possible explanation for this discrepancy between both studies could be the different patient population. DeCobelli et al. evaluated heterogeneous groups including non-ischemic heart-diseases. It is well-known, that the quantification in this more diffuse fibrosis is on one hand more challenging due to the small size and the blurred borders, but on the other hand the location in non-ischemic disease is typically intramural. That facilitates the differentiation from the bright blood pool signal. The different character of the fibrotic tissue in non-ischemic and ischemic heart disease seems to lead to the application of different standard-deviation for scar-sizing. In case of non-ischemic heart disease, often two standard deviations were applied to differentiate LGE from remote myocardium [18, 19]. For clinical decision making usually visual assessment of LGE images is recommended, whereas quantitative analysis of LGE extent and/or “grey-zone” extent is common for research purposes [20]. The integration of pharmacokinetic models with different compartments could also help to reduce the influence of different contrast dynamics in different types of fibrosis [21], as well as different analysis tools [22].

One would assume that parametric mapping techniques will help to overcome that problem. Whereas mapping techniques are already applied in different diseases and also for the quantification of extracellular volumes [23, 24], no standardized approach is given today. Furthermore automatic assessment of infarct borders will help to overcome subjective approaches [25], but they also depend on the predefined gold standards.

Independent of the quantification method itself, contrast media with higher relaxivity are warranted for potential dose reduction. The concept of dose reduction in contrast agents with higher relaxivity was also analyzed for gadobenate dimeglumine. Recent studies have shown a similar diagnostic performance and delineation of infarct scars using reduced doses of gadobenate dimeglumine compared to standard dose Gd-DTPA (0.2 mmol/kg BW) [26–28]. When comparing equivalent doses of gadobenate dimeglumine with Gd-DTPA [29], higher SNR- and CNR between scar and remote myocardium was observed for gadobenate dimeglumine, whereas the CNR between scar and blood decreased resulting in poorer delineation of small subendocardial infarcts. New technical developments could help to overcome this limit. Recently, a new multi contrast LGE sequence (MCODE) was proposed to improve detection of subendocardial myocardial infarction [30].

Reduced dose of gadobutrol allows a reproducible detection of myocardial infarction in all patients. In clinical routine visual assessment of scar is accepted and eye balling is accepted [12]. But scar quantification may play an important role in treatment planning in future and is expected to play a crucial role in risk stratification [1, 31, 32]. Therefore, a simple and robust post-processing method is required. The present study demonstrated that the inter-observer variability of LGE quantification by using the 6-SD threshold yielded satisfactory results in ischemic lesions independent from the contrast type and dose. However, we observed that relaxivity and dose influence the absolute results, which underlines that accurate follow-up evaluations need constant CMR conditions in clinical trials. CMR offers the unique capability to bring reliable (semi)quantitative approaches of myocardial tissue differentiation based on contrast- and non-contrast enhanced techniques into clinical routine therefore standardization is of importance.

## Limitations

The present study was conducted at a single center with a relatively small sample size. The study only included men to omit the influence of sex on the results. Further studies in women are necessary to extend the conclusions to both sexes.

SNR- and CNR of LGE images are influenced by inversion time. Its adjustment is operator-dependent. T1-mapping was expected to add information on contrast-media timing, but for technical reasons we were only able to complete a pairwise T1 analysis in six patients of group A. Larger studies preferably with mapping techniques are needed to confirm our results.

## Conclusions

The use of 0.15 mmol/kg BW gadobutrol led to comparable results as 0.20 mmol/kg BW Gd-DTPA in delineation of myocardial infarction and only to a small, clinically non-relevant deviation in quantification of infarct scar and may be an appropriate alternative under consideration of the lower risk for NSF. In contrast, the dose of 0.10 mmol/kg BW gadobutrol was associated with lower signal intensity and higher discrepancy regarding infarct size. Our results underline that accurate scar evaluation during follow-up depends on type of contrast agent and contrast dose and therefore requires constant study conditions.
